# Quantification of Specific Antibodies Against SARS-CoV-2 in Breast Milk of Lactating Women Vaccinated With an mRNA Vaccine

**DOI:** 10.1001/jamanetworkopen.2021.20575

**Published:** 2021-08-11

**Authors:** Erika Esteve-Palau, Araceli Gonzalez-Cuevas, M. Eugenia Guerrero, Clara Garcia-Terol, M. Carmen Alvarez, David Casadevall, Vicens Diaz-Brito

**Affiliations:** 1 Department of Infectious Diseases, Parc Sanitari Sant Joan de Déu, Sant Boi, Barcelona, Spain; 2 Department of Microbiology, Parc Sanitari Sant Joan de Déu, Sant Boi, Barcelona, Spain; 3 Department of Obstetrics and Gynecology, Parc Sanitari Sant Joan de Déu, Sant Boi, Barcelona, Spain; 4 Cancer Research Program, Hospital del Mar Research Institute, Barcelona, Spain

## Abstract

This cohort study assesses the concentration of SARS-CoV-2 antibodies in the breast milk of women who received vaccines for COVID-19 and their correlation with serum antibody levels.

## Introduction

The COVID-19 pandemic has raised questions among individuals who are breastfeeding, both because of the possibility of viral transmission to infants during breastfeeding and, more recently, of the potential risks and benefits of vaccination in this specific population. Previous studies have reported the presence of anti–SARS-CoV-2 antibodies in breast milk of COVID-19–infected lactating women,^1^ and recently several studies have demonstrated the passage of postvaccine antibodies through breast milk in women vaccinated with novel mRNA-based vaccines.^1,2^ In the present study, conducted between February and March 2021 at Parc Sanitari Sant Joan de Déu, an urban hospital in Spain, we sought to characterize the levels of specific SARS-CoV-2 antibodies in the breast milk of mRNA-vaccinated women across time, as well as their correlation with serum antibody levels.

## Methods

This prospective cohort study, carried out according to the Strengthening the Reporting of Observational Studies in Epidemiology (STROBE) reporting guideline, included lactating women older than 18 years who were vaccinated against SARS-CoV2 with the Pfizer-BioNTech COVID-19 vaccine. The ethics committee of the Sant Joan de Déu Research Foundation approved this study, and all participants signed for informed consent.

Serum and breast milk samples were simultaneously taken from each participant at 3 time points: 2 weeks after receiving the first dose of the vaccine (time point 1), 2 weeks after receiving the second dose (time point 2), and 4 weeks after the second dose (time point 3). All participants underwent nasopharyngeal SARS-CoV-2 rapid antigen testing (Ag-RDT) (Architect, Abbott). Levels of immunoglobin (Ig) G antibodies against the spike protein (S1 subunit) and against the nucleocapsid (NC) of SARS-CoV-2 were determined for each sample. Because vaccination does not induce nucleocapsid antibodies response, any IgG-NC positive result was considered as a prior infection. Statistical analyses were performed with R version 4.0.3 (R Project for Statistical Computing), and figures created using ggplot2 R package.

## Results

This study included 33 participants; mean (SD) age and postpartum time were 37.4 (3.3) years and 17.5 (10.1) months, respectively. No participants had confirmed SARS-CoV-2 infection prior to vaccination, nor during the study period (ie, tests for IgG-NC and Ag-RDT were all negative). We collected and analyzed 93 serum and milk samples from the 33 participants. Samples from time point 1 were taken at a median (range) of 14 (12-17) days after the first dose, while samples of time points 2 and 3 were taken at 14 (14-15) days and 28 (28-30) days after the second vaccine dose, respectively.

**Figure 1.**
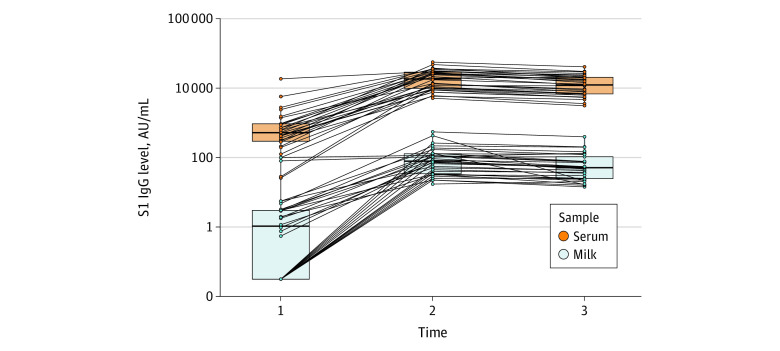
Evolution of Immunoglobulin (Ig) G S1 Subunit (S1) Levels in Breast Milk and Serum of Vaccinated Participants Across Time AU indicates arbitrary units.

**Figure 2.**
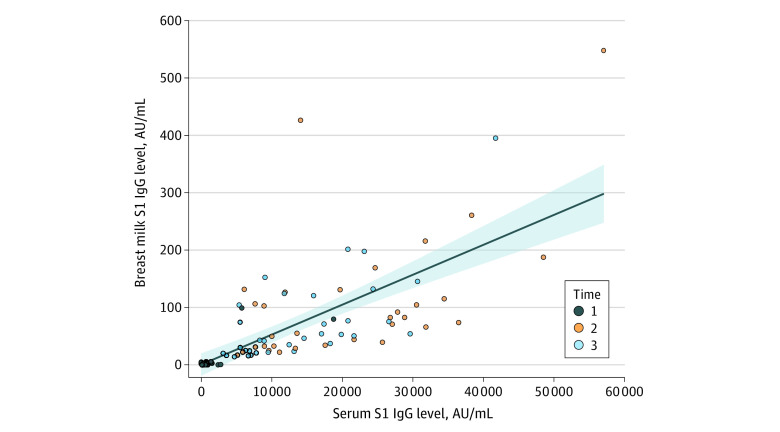
Correlation Between Immunoglobulin (Ig) G S1 Subunit (S1) Levels in Serum and Breast Milk of Vaccinated Participants AU indicates arbitrary units.

Median (interquartile range) IgG(S1) levels for serum–milk pairs at each time point were 519 (234-937) to 1 (0-2.9) arbitrary units (AU) per mL for time point 1, 18 644 (9923-29 264) to 78 (33.7-128) AU/mL for time point 2, and 12 478 (6870-20 801) to 50.4 (24.3-104) AU/mL for time point 3 (Figure 1). The Pearson correlation coefficient between breast milk and serum IgG(S1) levels was 0.7 (Figure 2).

## Discussion

Our results suggest that breast milk from women vaccinated with the novel mRNA-based Pfizer-BioNTech vaccine contains specific anti–SARS-CoV-2 IgG(S1) antibodies. Furthermore, we found that after the second dose, breast milk IgG(S1) levels increased and were positively associated with corresponding serum levels.

The main limitation of this study is its small sample size. It remains to be determined if breast milk antibody levels decrease or plateau after vaccination, or whether these findings can be reproduced for other mRNA and non–mRNA-based vaccines. The kinetics of IgG and other specific immunoglobulins against SARS-CoV-2, such as IgA and IgM, have been well studied after the disease^5^ (mainly in serum but also in breast milk^6^), although their dynamics after vaccination are not fully known. Larger prospective studies examining these issues are needed to confirm the safety of SARS-CoV-2 vaccination in individuals who are breastfeeding and further assess the association of vaccination with infants’ health and SARS-CoV-2-specific immunity.
